# Downregulation of tumor suppressive microRNAs *in vivo* in dense breast tissue of postmenopausal women

**DOI:** 10.18632/oncotarget.20906

**Published:** 2017-09-15

**Authors:** Annelie Abrahamsson, Alessandra Capodanno, Anna Rzepecka, Charlotta Dabrosin

**Affiliations:** ^1^ Department of Oncology and Department of Clinical and Experimental Medicine, Linköping University, Linköping, Sweden; ^2^ Department of Radiology and Department of Medical and Health Sciences, Linköping University, Linköping, Sweden

**Keywords:** mammary gland, microdialysis, mammography, extracellular miRNA, inflammation

## Abstract

Women with dense breast tissue on mammography are at higher risk of developing breast cancer but the underlying mechanisms are not well understood. De-regulation of microRNAs (miRNAs) has been associated with the onset of breast cancer. miRNAs in the extracellular space participate in the regulation of the local tissue microenvironment.

Here, we recruited 39 healthy postmenopausal women attending their mammography-screen that were assessed having extreme dense or entirely fatty breasts (nondense). Microdialysis was performed in breast tissue and a reference catheter was inserted in abdominal subcutaneous fat for local sampling of extracellular compounds. Three miRNAs, associated with tumor suppression, miR-193b, miR-365a, and miR-452 were significantly down-regulated in dense breast tissue compared with nondense breast tissue. In addition, miR-452 exhibited significant negative correlations with several pro-inflammatory cytokines *in vivo*, which was confirmed *in vitro* by overexpression of miR-452 in breast cancer cells. No differences were found of miR-21, -29a, -30c, 146a, -148a, -203, or -451 in breast tissue and no miRs were different in plasma. Extracellular miRNAs may be among factors that should be included in studies of novel prevention strategies for breast cancer.

## INTRODUCTION

Breast cancer is the most common cancer in women in the Western World, more than 10% of all women will be affected, and the incidence is still increasing [1]. As a result of mammography screening programs and improved treatments the death rate of breast cancer has declined [1]. Effective breast cancer prevention strategies would, however, lead to greater advances in breast cancer mortality and morbidity.

De-regulation of microRNAs (miRNAs), small endogenous non-coding molecules that regulate transcription of numerous genes, has been associated to onset and progression of various pathological processes including breast cancer [2–4]. Circulating miRNA in serum/plasma has been proposed as screening biomarkers and suggested to be therapeutic targets for clinical interventions [2–4]. There are, however, inconsistencies between studies, one reason being that no individual or sets of miRNAs have been validated to be invariant and suitable as endogenous controls [2–4]. In addition, blood miRNA reflects the net amount in the body rather than extracellular miRNA from a specific tissue. We have recently shown that several extracellular miRNAs exhibit a tissue specific expression in breast cancers and normal human breast tissue *in vivo*, differences that were undetectable in plasma [5].

Mammographic density has been associated with increased risk of breast cancer; women with increased dense breast tissue area on mammography compared to women with entirely fatty breast tissue (nondense) have a four-fold increased risk of developing breast cancer [6]. Several histological differences between dense and nondense breast tissue have been determined including higher amounts of stroma and less fat tissue in dense breasts [7]. Regarding the amount of epithelial cells, the proliferation rate, and steroid receptor expression conflicting results have been obtained [7]. Exposure to endogenous and exogenous sex steroids are established risk factors for breast cancer and anti-estrogen therapies reduce the risk of breast cancer by approximately 50% [8]. However, severe side-effects as well as decreased quality of life and low adherence of the therapeutics are associated with these treatments [8, 9]. Moreover, no associations have been found between circulating endogenous estrogen levels and breast density [7]. Thus, a more sophisticated biological characterization of dense breast tissue is key for the development of preventive measures of breast cancer associated with less toxicity compared to anti-estrogens. We have recently shown that dense breast tissue is associated with a pro-inflammatory microenvironment [10].

In this explorative study, healthy postmenopausal women attending the regular mammography-screening program with different breast densities were identified and microdialysis was performed for sampling of extracellular miRNAs and cytokines *in vivo*. We have previously shown that several extracellular *in vivo* miRNAs, sampled using microdialysis, were tissue specific expressed in breast cancers and normal breast tissue [5]. Therefore, we focused our attention to these miRNAs in the present study. We found that several extracellular miRNAs associated with tumor suppression were down-regulated in dense breast tissue and that miR-452 exhibited a negative correlation with several pro-inflammatory cytokines *in vivo*.

## RESULTS

### Subject characteristics

No statistical differences in age, body mass index (BMI), years since menopause, plasma levels of estradiol, or local extracellular breast estradiol levels were detected between the groups. Median and (range) were; for age 67 years (58-73) *vs.* 63 years (55-74), BMI 25 (19-30) *vs*. 24 (19-32), years since menopause 13 (6-22) *vs*.11 (3-21), plasma estradiol 112 pmol/l (22-184) *vs*. 126 pmol/l (4-175) in nondense *vs*. dense groups respectively. The local extracellular levels of estradiol in breast tissue were 38 pmol/l (22-69) in the nondense breast *vs*. 43 pmol/l (14-74) in the dense breasts.

There were no subsequent complications after the microdialysis investigations.

### Microdialysates contained exosomes

**Figure 1:**
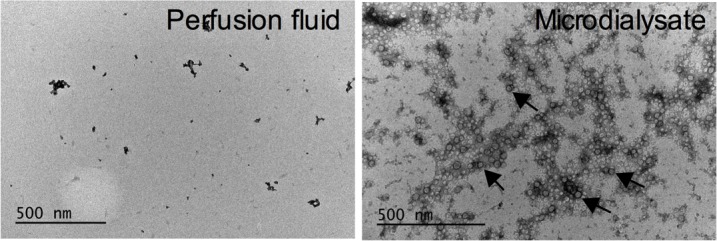
Microdialysis samples contain exosomes Electron microscope images of microdiaysis sample and control (perfusion fluid). Arrows indicate exosomes.

We have previously shown that two of the main binding proteins for miRNAs can be found in microdialysates [5]. miRNAs are also transported in membrane bound particles up to 1 μm in size or in exosomes, which previously have been suggested to be 50-90 nm in diameter [11, 12]. The pore size of the microdialysis membrane would exclude such large compounds. However, others have shown that exsomes may be considerably smaller, less than 10 nm [13, 14] and such small exosomes would theoretically pass the microdialysis membrane. To investigate if exosomes could be found in microdialysates, we performed electron microscopy of the microdialysates. Indeed, we found relatively large amounts of exosomes in the microdialysis samples, Figure 1. This was further confirmed by quantification of the exosomal marker CD9, which revealed that exosomes were present at 3.8×10^7^±7×10^5^ exosomes/μl microdialysate.

### Significant decreased expression of miR-193b, miR-365a, and miR-452, in dense breasts

**Figure 2:**
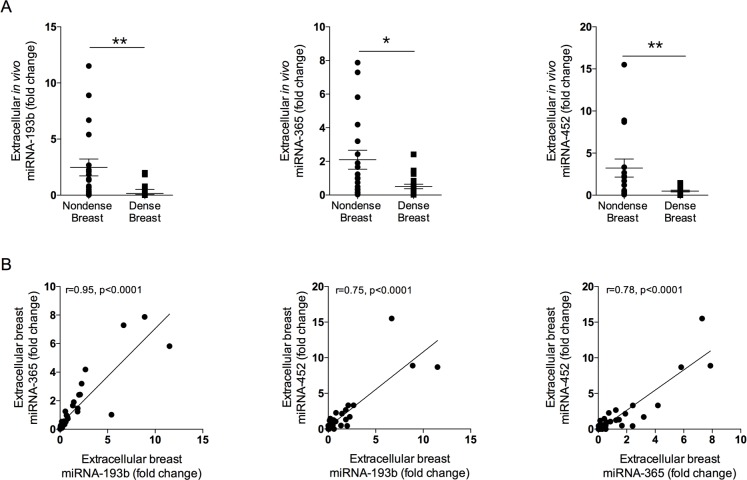
Expression of extracellular local miRNAs in normal breast tissue of various densities Thirty-nine healthy postmenopausal women, attending their regular mammography screen categorized as having either dense or nondense breast tissue underwent microdialysis as described in the materials and methods section. **(A)** Significantly decreased expression of miR-193b, -365a, and -452 were detected in dense breast tissue as compared with nondense breast tissue. Aligned dot plots with mean±SEM are depicted, ^*^p<0.05, ^**^p<0.01. **(B)** Significant positive correlations between the decreased miRNAs were found.

Significant decreased *in vivo* expression of extracellular miR-193b, miR-365a, and miR-452 were found in dense breast tissue compared to nondense breasts, Figure 2A. Significant positive correlations were also detected of these miRNAs, Figure 2B. No correlations were found between estradiol levels and the miRNAs.

No significant different expression levels of miR-21, -29a, -30c, -146a, -148a, -203, and -451 were found between the two different breast tissue densities, Supplementary Figure 1. No significant different levels of plasma miRNAs were found between the groups, Supplementary Table 1.

### Negative correlations between miR-452 and pro-inflammatory cytokines

Inflammation is one of the hallmarks of cancer progression [15]. We have recently shown that dense breast tissue is associated with a pro-inflammatory microenvironment [10]. Therefore we investigated if the miRNAs that were down-regulated in dense breast tissue exhibited any correlations with inflammatory cytokines. As shown in Table 1, no associations were found between miR-193b and -365a and the cytokines. However, miR-452 exhibited a significant negative correlation with the IL-1Ra/IL-1β ratio, IL-8, CCL5, and VEGF, Table 1.

**Table 1:** Pro-inflammatory cytokines exhibit significant negative correlations with miR-452 in normal human breast tissue

Variable	IL1Ra/IL-1β	IL-6	IL-8	CCL2	CCL5	VEGF
miR-193b	0.16	-0.07	-0.25	0.09	-0.08	-0.19
miR-365a	0.20	0.02	-0.20	0.21	-0.14	-0.16
miR-452	0.39^*^	-0.23	-0.56^***^	0.059	-0.41^*^	-0.44^**^

^*^ *p*<0.05, ^**^p<0.01, ^***^p<0.001.

Spearman’s Correlation Coefficients of extracellular *in vivo* miRs (fold change in breast tissue compared to controls in subcutnaeous abdominal fat tissue) and extracellular *in vivo* concentrations of cytokines in normal human breast tissue, n=39. Significant values in bold.

### Significant alternations of secreted pro-inflammatory cytokines after transfection of miR-452 into breast cancer cells

**Figure 3:**
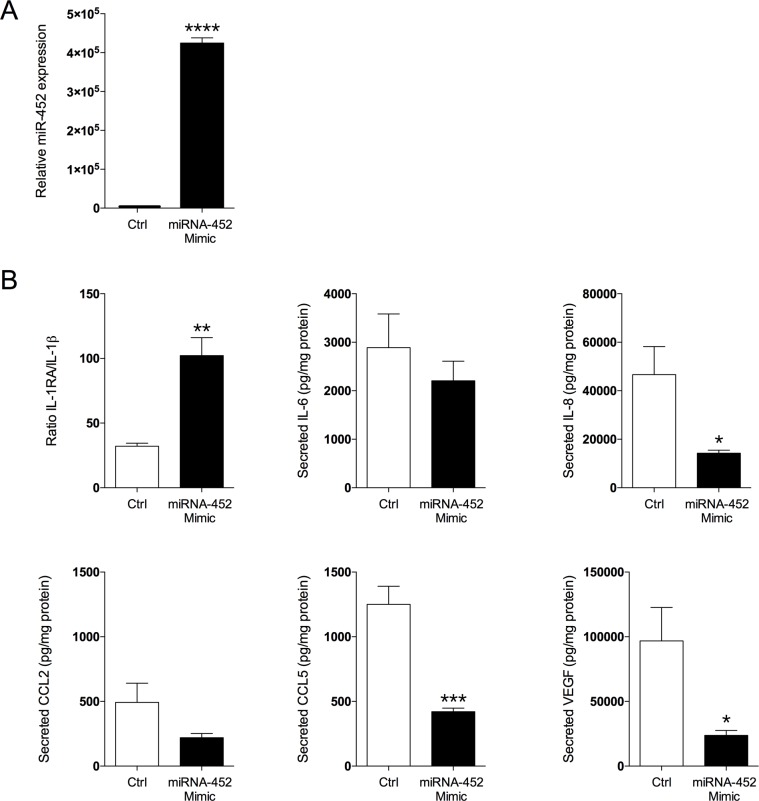
Transfection of miR-452 to breast cancer cells resulted in altered secretion of cytokines MDA-MD-231 breast cancer cells were transfected with miR-452 or its control as described in the materials and methods section. **(A)** Transfection resulted in increased the expression of miR-452, ^****^p<0.0001. **(B)** Levels of secreted cytokines after miR-452 transfection, n=5 in each group, ^*^p<0.05, ^**^p<0.01, ^***^p<0.001. Bars represent mean±SEM.

To elucidate if miR-452 directly affects the inflammatory profile we set-up *in vitro* culture of breast cancer cells and transfected them with miR-452mimic. Secreted levels of the cytokines were thereafter quantified. Indeed, our *in vivo* results were corroborated by the *in vitro* experiments. As shown in Figure 3 over-expression of miR-452 resulted in a significant decreased secretion of IL-8, CCL5, and VEGF and an increased ratio of IL-1Ra/IL-1β, n=5 in each group.

## DISCUSSION

Here we show that dense breast tissue in postmenopausal women exhibits decreased expression of several extracellular miRNAs that have been associated with tumor suppression. The *in vivo* extracellular expression levels of miR-193b, miR-365a, and miR-452 were significantly decreased in dense breast tissue compared to nondense breast tissue. None of these changes were detected in plasma, suggesting that these alterations were breast specific. In addition, miR-452 exhibited significantly negative correlations with pro-inflammatory cytokines *in vivo* and these results were corroborated by overexpression of miR-452 into breast cancer cells *in vitro*. No differences were found of expression levels of miR-21, miR-29a, miR-30c, miR-146a, miR-148a, miR-203, or miR-451 between dense and nondense breast tissue or in plasma. Additionally, we show that exosomes can be collected *in situ* with the microdialysis technique.

In breast cancer, cellular miR-193b has been shown to be down-regulated but to our knowledge, no reports in circulating miR-193b have been published [16]. In breast cancer cell lines as well as in murine breast cancer models, reconstitution of miR-193b resulted in reduced proliferation, migration, invasion, and dissemination supporting miR-193b as a tumor suppressor [17, 18]. These studies were performed in estrogen receptor (ER) positive as well as in ER negative cancer cells [17, 18]. In experimental models, miR-193b may also interact with enzymes involved in local estrogen production resulting in decreased estradiol levels [19]. We have previously shown that extracellular breast miR-193b was significantly lower in postmenopausal breast tissue compared with premenopausal breasts supporting a link between miR-193b and estrogen levels [5]. However, no correlations with miR-193b and local breast estradiol levels were detected in the current study suggesting that other factors than miR-193b may be more important for local estrogen production.

miR-193b is co-located with miR-365a on chromosome 16 and these two miRNAs have been shown to cluster in adipose tissue as well as in epidermal squamous cell carcinoma [20, 21]. We found that miR-365a was significantly down-regulated in dense breast tissue and that miR-193b and miR-365a correlated significantly supporting previous findings of a co-expression of these miRNAs also in the extracellular space.

Cellular miR-365a has been shown to be tumor suppressive in several cancer forms [22, 23]. Similar to miR-193b, miR-365 has been shown to exert its tumor suppressing effects mainly by decreasing breast cancer cell proliferation [24, 25]. Moreover, a tumor suppressive effect in breast cancer is supported by clinical data where it has been demonstrated that circulating miR-365 in blood is down-regulated in breast cancer patients [26, 27].

Regarding circulating miR-452 and breast cancer the literature is sparse. Cellular expression of miR-452, however, has been associated with tumor suppression; in breast cancer tissue, miR-452 has been shown to be down-regulated whereas no differences were found in serum from healthy volunteers and metastatic breast cancer patients [28]. Down-regulation of miR-452 has also been shown to promote stem-like traits, progression, and migration of several cancer types [29–32]. A potential relationship between miR-452 expression and inflammatory response has previously not been investigated. However, miR-452 is predicted to target *IL-8* gene by binding to a complementary region in the 3′UTR at the position 711-717. We have recently shown a pro-inflammatory microenvironment in dense breast tissue of postmenopausal women [10] and we show in the present data significant negative correlations between miR-452 and several pro-inflammatory cytokines. These results were corroborated in vitro, where over expression of miR-452 in breast cancer cells resulted in decreased secretion of the same cytokines. To the best of our knowledge, this is the first study deciphering this regulation. Thus, in addition to inhibition of cell proliferation, migration, and invasion, miR-452 may also play a role in the modulation of the inflammatory response.

In addition to decreased expression of miR-193b, miR-365a, and miR-452 in dense breast tissue the levels of these miRNAs exhibited highly statistical significant positive correlations.

Blood levels of miRNAs have been suggested as useful biomarkers of cancer progression but there is a lack of consistency between different studies [2–4]. The major problems are a lack of standardized protocols for sample preparation and a lack of extracellular validated endogenous controls, therefore a fixed input volume has been suggested to be the most suitable constant for extracellular miRNAs [33]. We have previously used this approach in combination with absolute quantifications using standard curves and showed that extracellular miRNAs in microdialysis samples are stable and exhibit very low extraction variability [5]. The same approach was used in the present study. To overcome the issues with endogenous controls we used the extracellular levels of the specific miRNA in abdominal subcutaneous fat for normalization of the expression levels in the breast and calculated the relative expression levels of individual miRNAs in breast tissue.

It is clear that local sampling of extracellular miRNAs in the tissue of interest gives further insights in the local tissue microenvironment as no differences of plasma levels of miRNAs between the groups were detected in our study.

A better understanding of the molecular mechanisms associated with breast carcinogenesis in dense breast tissue is a prerequisite for finding proper prevention strategies. Extracellular miRNAs are important cell-cell signaling transmitters regulating the tissue microenvironment. Our data suggest that extracellular miRNAs are among the factors that should be included in studies of the biology of breast tissue with increased risk of breast cancer.

## MATERIALS AND METHODS

### Subjects

The study was performed in accordance with the Declaration of Helsinki and the regional ethical review board of Linköping approved the study. All women gave informed written consent. Thirty-nine postmenopausal healthy women (55 years of age or older) were recruited from the screening mammography program at Linköping University Hospital. Exclusion criteria were previous breast cancer, current use of hormone replacement therapy, any clotting or metabolic disorder, or use of non-steroidal anti-inflammatory drugs (NSAIDs). All of the women were non-smoking. The mammographic density was assessed using the Breast Imaging Reporting and Data System (BI-RADS) density scale [34] by one experienced observer (AR). Women with breast densities categorized as BI-RADS A, (entirely fatty nondense breasts), or BI-RADS D (extremely dense) were identified.

### Microdialysis procedure

Prior to insertion of the microdialysis catheters, 0.5 ml lidocain (10 mg/mL) was administrated intracutaneously. One microdialysis catheter was placed in the upper lateral quadrant of the left breast and directed towards the nipple and another in abdominal subcutaneous fat as previously described [5, 35–40]. Microdialysis catheters (#71, M Dialysis AB, Stockholm, Sweden), which consist of a tubular dialysis membrane (length 20 mm, diameter 0.52 mm, 100,000 atomic mass cut-off) glued to the end of a double-lumen tube (80 mm long x 0.8 mm in diameter), were inserted via a splitable introducer (M Dialysis AB), connected to a microinfusion pump (M Dialysis AB) and perfused with NaCl 154 mmol/L and hydroxyethyl starch 60g /L (Voluven®, Fresenius Kabi, Uppsala, Sweden), at a perfusion rate of 0.5 μL/min. After a 60-min equilibration period, the outgoing perfusate was stored at –70°C. EDTA plasma was collected and stored at –70°C.

### Extracellular miRNA extraction

The ZR RNA MicroPrep kit (Zymo Research Corporation, CA, USA) was used for miR extraction from 30 μl microdialysate or 80 μl EDTA plasma and eluted in 6μl nuclease-free water. A spike in control, *Caenorhabditis elegans* miR-67-3p (Life technologies, CA, USA), was added prior extraction.

### Reverse transcription, pre-amplification, and real-time quantitative PCR (RT-qPCR) of extracellular miRNA

TaqMan MicroRNA Reverse Transcription Kit (Life technologies, CA, USA) was used for 3 μl of extracted miRNAs. Pre-amplification of converted DNA (cDNA) was performed with TaqMan PreAmp Master Mix (2X) (Life technologies, CA, USA) and Megaplex PreAmp Primers (10X) (Life technologies, CA, USA). RT and pre-amplification were run on ProFlex™ PCR System (Life technologies, CA, USA). RT-qPCR for mature miRNAs was performed using 1 μl of the pre-amplificated cDNA product. Each miRNA was run in duplicate using the 7900HT Fast Real-Time PCR System from Applied Biosystems, MS,USA.

### Absolute quantification of extracellular miRNAs

Synthetic miRNA mimic (Ambion) representing the mature miR sequences (miRBase v.21), were used to generate standard curves for each miRNA. RT and pre-amplification were performed similar to the microdialysis and plasma samples. A line was fit from each dilution using threshold cycle (C_t_) values and the concentration of miRNA was converted to fmol/l.

As there are no validated endogenous control or sets of endogenous controls for extracellular miRNA we chose to use a fixed volume as an input in the analyses as recently described [5, 33]. Thereafter, absolute quantifications using standard curves were performed and the levels in microdialysis samples from abdominal subcutaneous fat were used for normalization of each individual miRNAs in the breast. Thus, the levels in breast tissue are expressed as fold change compared to the levels in abdominal subcutaneous fat tissue.

### Cell culture and transfection

The human breast cancer cell line MDA-MB-231 was obtained from the American Type Culture Collection (Rockville, MD, USA) and authenticated using the short tandem repeat profiling at the Uppsala Genome Center. The cells were cultured in Dulbecco’s modified Eagle medium (DMEM) supplemented with 10% fetal bovine serum and 2 mM L-glutamine, and maintained at 37°C in a humidified atmosphere with 5% CO_2_. 2×10^5^ cells/well in 6-wells plates were seeded and the next day, cells were transfected for 48 hours with 25 nM mirVana™ miR Mimic Negative Control #1 (Ctrl) or 25 nM mirVana™ miR Mimic hsa-miR-452 using Lipofectamine® RNAiMAX according to the instructions provided by the manufacturer. All the reagents were from Life Technologies (CA, USA).

### Intracellular miRNA quantification

To assess the transfection efficiency, RT-qPCR analysis was used to determine the expression level of the mature miR-452 and U6 snRNA as endogenous control in MDA-MB-231 cells. Total RNA was isolated using the mirVana™ miRNA Isolation Kit (Life Technologies) according to the manufacturer’s instructions. The quantity and purity of the isolated RNA were assessed using Nanodrop ND-1000 spectrophotometer (Thermo Scientific, MS, USA). Total RNA (10 ng) was retro-transcribed using the TaqMan^®^ MicroRNA Reverse Transcription Kit (Life technologies, CA, USA) using the ProFlex™ PCR System (Life technologies, CA, USA), and 1 μl of cDNA was analyzed by RT-qPCR using 7900 HT Fast Real-Time PCR System (Applied Biosystems, MS, USA). All samples were assessed in duplicate and miR-452 relative expression was determined by the 2^−ΔΔCt^ method.

### Cytokine and estradiol quantification

After 48 hours of transfection, the medium was removed and cells were cultured in DMEM complete medium for additional 24 hours. Thereafter the medium was collected and stored at -80°C until analyses. The medium was analyzed for CCL5 and IL-1Ra (Quantikine ELISA), and IL-1β, IL-6, IL-8, and VEGF (QuantiGlo ELISA), and CCL2 with Luminex® Performance Assay using the Luminex 200 system (Luminex Corporation, TX, USA). All the assays were purchased from bio-techne (Abingdon, UK). Total protein content of the cell pellets were determined by Pierce™ BCA Protein Assay (Thermo Fisher, MS, USA) according to the manufacturer’s instructions. The cytokine concentration was normalized against the total protein concentration in the cell lysate. Cytokines in the microdialysates were analyzed using Human Fluorokine MAP kits with corresponding beads on a Luminex 200 System (Luminex, Austin, TX). Estradiol was quantified using an EIA-kit (DRG Diagnostics, Marburg, Germany).

### Exosome isolation and quantification

Exosomes were isolated from microdialysate using the ExoQuick-TC precipitation solution (System Biosciences, CA, USA). The microdialysates were centrifuged at 3000 xg for 15 minutes and the ExoQuick-TC precipitation solution was added to the supernatant at 1:5 and incubated overnight at 4°C. Thereafter, the mixture was centrifuged at 1500 xg for 30 minutes at 4°C and then at 1500 xg for 5 minutes to remove all traces of fluid. The number of exosome particles was quantified by using the CD9 ExoELISA™ kit (System Biosciences, CA, USA) according to the manufacturer’s instruction.

### Exosome electron microscopy

Exosome electron microscopy was performed as described previously [41] with some modifications. Briefly, purified exosomes were resuspended in ice-cold PBS and transferred to Formvar-coated copper electron microscopy grids. Grids were negatively stained with 2% uranyl acetate and electron micrographs were obtained using a JEOL JEM1230 transmission electron microscope at 60-120 kV.

### Statistical analyses

Statistical analyses were performed using GraphPad Prism software 6.0. Two-tailed Student’s t-test or Spearman’s Correlation Coefficients were used where appropriate. Data are expressed as mean±SEM. A *p*<0.05 was considered as statically significant

## SUPPLEMENTARY MATERIALS FIGURE AND TABLE


