# Supplementary material for: Downregulation of tumor suppressive microRNAs in vivo in dense breast tissue of postmenopausal women
Source: Oncotarget. 2017 Sep 15;8(54):92134–42. doi: 10.18632/oncotarget.20906 (PMC5696169; doi:10.18632/oncotarget.20906)
Supplement: Supplementary file 1 [file oncotarget-08-92134-s001.pdf]

Downregulation of tumor suppressive microRNAs *in vivo* in dense breast tissue of postmenopausal women

SUPPLEMENTARY MATERIALS

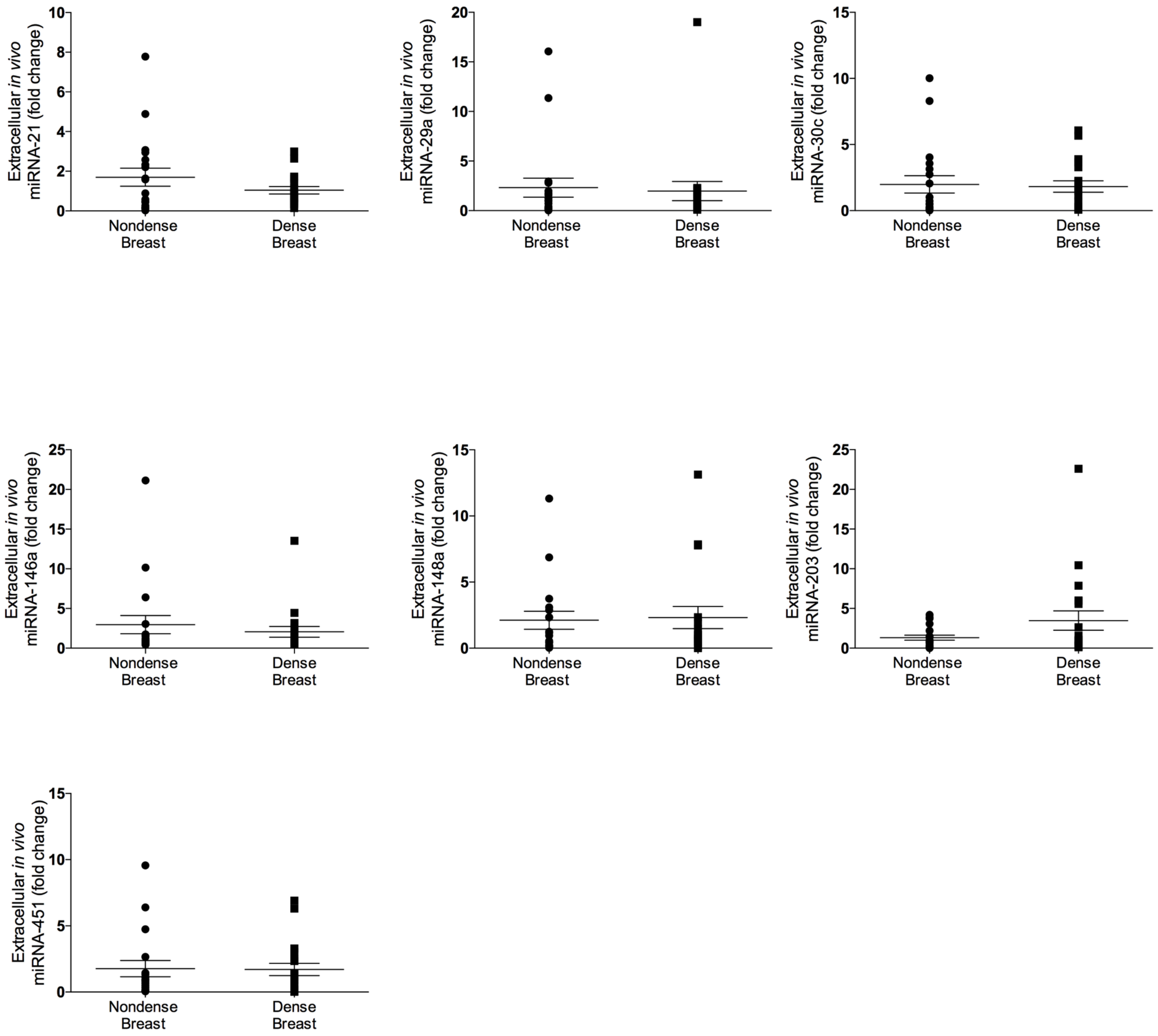

Supplementary Figure 1: No significant differences of miR-21, -29a, -30c, -146a, -148a, -203, or -451 were found in dense versus nondense breast tissue of postmenopausal women. Thirty-nine healthy postmenopausal women, attending their regular mammography screen categorized as having either dense or nondense underwent microdialysis as described in the materials and methods section. Aligned dot plots with mean±SEM are depicted.

Supplementary Table 1: Plasma levels of microRNAs in postmenopausal women with dense or entirely fatty breasts (nondense) on their regular mammography screen expressedas fmol/l ±SEM

	Nondense (n=19)	Dense (n=20)	*p*-value
miR-21	88131±15805	116545±13161	0.2
miR-29a	21760±4232	20831±3070	0.9
miR-30c	152523±24730	171765±16593	0.5
miR-146a	$1.6x10^8 \pm 8x10^7$	$0.7x10^8 \pm 7x10^7$	0.4
miR-148a	2544±420	3776±553	0.1
miR-193b	15087±2533	18651±2741	0.4
miR-203	885±167	10616±9750	0.3
miR-365a	34048±5784	38166±4915	0.6
miR-451	$1.2x10^6 \pm 2x10^5$	$1.9x10^6 \pm 3x1^5$	0.1
miR-452	395±64	446±112	0.7
